# The Work and Duties of the Ohio State Dental Society

**Published:** 1892-04

**Authors:** W. H. Sedgwick


### THE DENTAL REGISTER.

VOL. XLVI.] APRIL, 1892. [No. 4.

### COMMUNICATIONS.

### The Work and Duties of the Ohio State Dental Society.

BY W. H. SEDGWICK, D. D. S.

Read before the Ohio State Dental Society, Dec. 3, 1891.

The Dental Societies and our Dental literature have in my remembrance, taken up from time to time different subjects connected with our specialty, and discussed and written, and rewritten on them until the whole subject seemed to have been exhausted, and ourselves in danger of being charged with riding a hobby. Some of these important subjects as they occur to me now, are the comparative value of soft and cohesive gold, contour fillings occupied our attention for several years, when irregularities crowded all other questions from our programmes; the cause of dental caries, dependent on chemical action attracted our attention for a while, when lo and behold the microscope of Doctor Miller brought to our notice the teeming millions of microbes and bacteria, with all their numerous relatives, which in time came in for a due share of our attention; when it dawned upon the savants, that these insidious enemies to our further success must be put to death, routed, and entirely exterminated, which has brought to our attention, and taxed to the utmost, the learned scientists of our specialty to discover the chemical, or combination of elements, heat or cold, that would accomplish the entire extermination of these infintesimal, insidious enemies, that had for so many years stood between us and success. Thus has the question of disinfectants and antiseptics been thrust upon us, and even now occupy the attention of the learned scientists of our specialty as witness one of the subjects on your present programme.

Now the free, full and almost exhaustive discussion of all and each of these questions in our Societies and Dental Journals has been of incalculable benefit and profit in the world of dental practice; and especially to the conscientious, industrious student of Dental literature.

In view of the fact that these important subjects have occupied the attention of the profession for so many years, and have in a measure been satisfactorily settled; it has occurred to me that it would be eminently proper and valuable, especially to the members of our profession in this State, that this State Society now take up with energy, and push to completion the enactment of a law by the legislature for the regulation of the practice of Dentistry in Ohio. The importance of this question is now especially brought to the attention of the people, by the unearthing and exposing by the Cincinnati *Commercial-Gazette* of the fraudulent issuing of Medical Diplomas in that city, and the action the medical profession of every school of medicine, Allopathic, Homœopathic and Eclectic have taken in the matter, suggests that this is an opportune time for this Society and the profession throughout the State to act with energy and promptness for a law regulating the practice of our specialty. As ours is the most important and perhaps the most extensively practiced of all the specialties of medicine, and perhaps more than any other by which the common people are being humbugged and injured, not excepting the mother profession itself. It is not necessary in my view of this subject that such a law should be so very lengthy and complicated, but simply providing that every person practicing Dentistry in this State shall have a diploma from some reputable Dental College of this or any other State. Such a diploma from such a school represents just so much study and careful preparation. Then every practitioner of Dentistry in this State should have a record made of such qualification in the county clerk's or recorder's office in every county where he expects to practice; I would also make it the duty of the prosecuting attorney of each county to bring suit against any one found or known to practice in his county without such record, this would remove the objection of one Dentist informing on one practicing contrary to law, and would in a great measure put a stop to the itinerancy now so prevalent throughout the State; and also to a "green" student after taking his first course in College establishing himself in some country town and advertising as " Doctor Blank, just from ——— Dental College," " all work warranted and teeth extracted without pain." Of course such a law would be incomplete without a just and suitable penalty attached.

If the straight and narrow path through the Dental College is to be the only *entree* to our profession, what! I hear you ask is to be done with our " *State Board of Examiners?*" My answer is, abolish it, it has outlived its usefulness. While it was eminently necessary and called for when organized, I can see no reason at this day why any should enter the door of our specialty by subjecting themselves to a casual examination on the general practice of dentistry and paying a fee of twenty-five dollars, while other young men, ambitious to take an honorable position in their profession spend the time necessary to complete a three years' course in a Dental College, at the expense of one thousand or twelve hundred dollars.

I am aware that some of our most excellent and honorable Dentists of today hold State certificates, but I am arguing now that from this on a diploma from some honorable, reputable Dental College should be a sufficient and the only passport to practice Dentistry; thus making the Dental College responsible if incompetent charlatans are preying on the incredulous and ignorant public. So I am opposed to the extra and expensive machinery of an Examining Board as advocated by some very eminent educators, both east and west. It is a reflection on the professors and teachers of our colleges, such incompetency, if any existed, would naturally be a proper question to come before the " National Association of Dental Colleges."

The only use then I can see for a State Board of Examiners would be to investigate the workings and methods of the Dental Colleges of our State, and to report to the proper county officials what colleges are legitimate and what diplomas should be received for record.

I wish to mention one element that will be brought to bear on the members of the legislature against the passage of a law for the regulation of the practice of medicine that perhaps has not been considered. The traveling specialists going from one county to another, pay the county editor more for advertising one week than all the regular practitioners of his town do in five years; consequently the county papers all over the State will oppose a law that will interfere with the traveling specialist; even though he is injuring and humbugging the general public. The publishers of our city papers make more money out of a small space in the lower right hand corner of the last column of their papers for one issue in advertising "secret remedies," "Manhood, how lost, how restored," etc., than they do out of the entire profession of their city for years.

The publishers will consequently oppose any law taking away from them this patronage, so a reputable editor and publisher of an influential county paper told me.

I expect criticism, for many of the views here advanced, and that is just what is desired, and will I hope attract the attention of those more able to discuss these questions than your humble servant, and result in upholding and advancing the standard of Dental practice.

